# Panhypopituitarism Presents As Amenorrhea Secondary to Post Traumatic Stress Disorder in a 33-Year-Old Patient: A Case Report

**DOI:** 10.7759/cureus.22812

**Published:** 2022-03-03

**Authors:** Yakubmiyer Musheyev, Maria Levada, Farage Ftiha, Iana Garrick, Habiba Ahasan, Matthew Jiang

**Affiliations:** 1 Medicine, New York Institute of Technology College of Osteopathic Medicine (NYITCOM), Old Westbury, USA; 2 Obstetrics and Gynaecology, New York Institute of Technology College of Osteopathic Medicine (NYITCOM), Old Westbury, USA

**Keywords:** hypopituitarism, hypothyroidism, post traumatic stress disorder, ptsd, amenorrhea, panhypopituitarism

## Abstract

Hormonal derangements should be suspected whenever a patient experiences amenorrhea with no abnormal physical exam findings. Clinical suspicion is increased if she also reports psychological trauma that could affect her nervous system and, by association, her hormones since the pituitary gland is present in the brain. Additional exams that aid in the diagnosis of amenorrhea include a variety of blood panels and imaging scans. Panhypopituitarism is a disorder in which there is a deficiency of all pituitary hormones that include but are not limited to the thyroid-stimulating hormone (FSH), luteinizing hormone (LH), and follicle-stimulating hormone (FSH). Diagnosis is usually made by baseline blood sampling of these hormones. Secondary to panhypopituitarism, amenorrhea can be disguised as other neurogenic problems. In this case study, we present a 33-year-old female patient who presented to the clinic with amenorrhea and a traumatic past social history. Upon further workup of the patient, it was determined that the patient had panhypopituitarism that had to be managed with medications indefinitely. This case study is of the utmost interest because it highlights how panhypopituitarism, being such a rare condition, can easily be mistaken as amenorrhea secondary to psychological issues and how integral it is for a physician to keep an open mind when evaluating such patients.

## Introduction

The menstrual cycle has been implicated as a sex-specific biological process influencing psychological symptoms across a variety of disorders [1]. One such disorder is posttraumatic stress disorder (PTSD), which is a disabling psychiatric disorder that results from being exposed to real or threatened injury, death, and sexual assault [2]. According to the Diagnostic and Statistical Manual of Mental Disorders (DSM-5), the diagnostic criteria for PTSD include but are not limited to stressors, intrusion symptoms, avoidance, and negative alterations in the mood [2]. PTSD is often associated with alterations in the hypothalamic-pituitary-adrenal (HPA) axis [3]. And it has been established that psychological stress also induces hypothalamic anovulation (amenorrhea). However, this type of anovulation is limited to the gonadotropin axis, and is commonly reversible after the source of stress is removed [4]. It should therefore come as no surprise that PTSD may have implications for causing hormonal imbalances among women, leading to all kinds of issues such as amenorrhea, weight loss, fatigue, vaginal discharge, and more stress. 

It is interesting to note that changes in the menstrual cycle are also implicated in another disorder, hypopituitarism. As opposed to panhypopituitarism, in which all pituitary hormones are deficient, hypopituitarism can present as a deficiency of individual anterior pituitary hormones (e.g., adrenocorticotropic hormone, thyroid-stimulating hormone (TSH), luteinizing hormone (LH), follicle-stimulating hormone (FSH), prolactin, growth hormone) or posterior pituitary hormones (e.g., oxytocin, vasopressin) [5]. Hypopituitarism is diagnosed based on baseline blood sampling for thyroid-stimulating hormone, gonadotropin, and prolactin deficiencies, whereas for adrenocorticotropic hormone (ACTH), growth hormone, and antidiuretic hormone deficiency, dynamic stimulation tests are usually needed; repeated pituitary function assessment at regular intervals is needed for diagnosis of the predictable but slowly evolving forms of hypopituitarism [6].

## Case presentation

A 33-year-old female Gravida 0 Para 0 presents to her OBGYN with complaints of irregular menses. During the last year, she has not had normal menses: her last menstrual period was 14 months prior to this visit. Prior to the visit, the patient had used a pregnancy test and it was negative. Her past medical history was significant for herpes simplex virus, leukopenia, and deep pain during intercourse. Her social history was significant for previous tobacco use and being deployed in Afghanistan for the past year; the patient is now a police officer. Because of her past military history, she suffers from PTSD. A physical exam of the abdomen, female genitalia, skin, neck, lungs, cardiovascular system, breast, and lymph nodes did not reveal any abnormalities; the patient had a BMI of 21.8 at the time of the visit. The patient was deemed stable. The differential diagnosis for a patient presenting with irregular menses with a history of PTSD is amenorrhea secondary to PTSD, idiopathic amenorrhea, and hypopituitarism.

On the first visit, the OBGYN ordered a thyroid panel, female hormone evaluation, lipid panel, and a CBC. The results revealed high serum cortisol levels as well as low serum levels for estradiol, FSH, LH, TSH, and T3. A pap smear was also done, and it came back negative for an intraepithelial lesion or malignancy. The CBC results were normal.

It is interesting to note that the HDL level in our patient was significantly lower. This is best explained by the fact that estradiol stimulates HDL production [7] and our patient had low estradiol levels due to the presumed diagnosis of hypopituitarism/panhypopituitarism. Furthermore, homocysteine levels were abnormally high. Previous literature [8] has established that homocysteine levels are elevated in hypothyroidism, which is seen in this patient who has low T3 and TSH levels.

In follow-up visits, these abnormalities stayed consistently out of range (Table 1). In short, the majority of the pituitary hormones were abnormally low, except for cortisol, which is produced by the adrenal glands. For a patient presenting with a deficiency of a collection of anterior pituitary hormones, the diagnosis is hypopituitarism and possibly panhypopituitarism.

**Table 1:** Comparing initial blood levels of hormones, enzymes, and lab values to normal ranges in a patient with panhypopituitarism Normal ranges based on Quest Diagnostic Incorporated Laboratories.

Hormone/enzyme/test	Patient’s value	Normal range
Follicular-stimulating hormone	<0.7 mIU/ml	2.5–9.1 mI/ml
Luteinizing hormone	<0.2 mIU/ml	0.5–76.3 mIU/ml
Thyroid-stimulating hormone	0.3 mIU/L	0.4–4.5 mIU/L
T3, total	49 ng/dL	76–101 ng/dL
Estradiol	<15 pg/ml	19–214 pg/ml
Cortisol	26.7 mcg/dL	3–17 mcg/dL
Homocysteine	136 µmol/L	<10.4 µmol/L
HDL	12 mg/dl	>50 mg/dl

The patient was further referred to an endocrinologist and the diagnosis of panhypopituitarism was confirmed. The patient was further educated about their condition. Eventually, prescriptions were given for estradiol, progesterone, and thyroid hormones to maintain optimal health. The patient was advised to get blood work routinely done (every three months) in order for pituitary hormone levels to be evaluated.

## Discussion

In this case report, we present a 33-year-old female who served in the United States military. Our patient had been deployed to Afghanistan for one year and now works as a police officer. The patient had a past medical history of irregular periods, painful intercourse, herpes simplex, and leukopenia. She also has PTSD because of her combat history, as previously mentioned. PTSD is often associated with alterations in the HPA axis [3]. And it has been established that psychological stress also induces hypothalamic anovulation (amenorrhea). However, this type of anovulation is limited to the gonadotropin axis and is commonly reversible after the source of stress is removed [4]. It is interesting to note that changes in the menstrual cycle are also implicated in other disorders such as hypopituitarism, which is a rare disorder in which the pituitary gland fails to produce one or more of its hormones. However, in a case where the pituitary gland does not produce any of its hormones, this would be diagnosed as panhypopituitarism and is extremely rare, occurring in 4.2 cases per 100,000 people per year [5].

It is worthwhile to mention that the physical examination of a patient with panhypopituitarism is often normal, and often there are minimal irregular findings. In this case, the physical exam of the patient was unremarkable and did not explain the patient’s chief complaint. The abnormalities were found when blood work was ordered. The blood work of the patient revealed that the patient had low levels of pituitary hormones except for cortisol, which is produced by the adrenal glands when under chronic stress. FSH, estradiol, LH, T3, TSH, and LDL levels were all below the normal physiologic range. Usually, bloodwork is the only way in which panhypopituitarism can be effectively diagnosed because bloodwork is the only way to evaluate the pituitary hormones.

The general treatment for panhypopituitarism is hormone replacement. Usually, patients diagnosed with this disease must take hormone supplements for the rest of their lives; they must also have regular blood tests in order to check the levels of said hormones. In this case, as previously mentioned, the patient was prescribed hormone supplements (such as estradiol, progesterone, and thyroid hormones) that required blood work to be routinely taken in order to confirm that the supplements were effective.

## Conclusions

It is not uncommon for patients with PTSD to present with amenorrhea. However, other disease processes could be present, as in the case of this patient, who was ultimately diagnosed with panhypopituitarism. This is why it is important for all clinicians to keep an open mind when seeing female patients who present with irregular menses.

To reiterate, the main takeaway of this study is that it is imperative for physicians to have a broad differential when seeing a patient coming in with complaints of irregular menses. For example, in this case, we have a female patient coming in with complaints of irregular menses for the past 14 months; this information, coupled with the fact that she has PTSD from being in the military during this time, could lead her physician to erroneously think her condition has to do something with her stress in the military. However, we see here that this is not the case, and this is why this study is of the utmost interest, for it shows that physicians have to look beyond the obvious. Ultimately, in this case study, we present a 33-year-old female patient who presented to the clinic with signs and symptoms of amenorrhea secondary to PTSD. Upon further workup of the patient, it was determined that the patient had panhypopituitarism.
